# Inflammatory mechanism of Rumenitis in dairy cows with subacute ruminal acidosis

**DOI:** 10.1186/s12917-018-1463-7

**Published:** 2018-04-19

**Authors:** Chenxu Zhao, Guowen Liu, Xiaobing Li, Yuan Guan, Yazhou Wang, Xue Yuan, Guoquan Sun, Zhe Wang, Xinwei Li

**Affiliations:** 1 Key Laboratory of Zoonosis, Ministry of Education, College of Veterinary Medicine, Jilin University, 5333 Xi’an Road, Changchun, Jilin, 130062 China; 2 College of Animal Science, Jilin University, 5333 Xi’an Road, Changchun, Jilin, 130062 China; 3 College of Animal Science and Technology, Inner Mongolia National University, Tongliao, 028000 Inner Mongolia China

**Keywords:** Subacute ruminal acidosis, LPS, Rumenitis, Inflammatory pathway

## Abstract

**Background:**

Subacute ruminal acidosis (SARA) is a metabolic disease in high-producing dairy cattle, and is accompanied by rumenitis. However, the mechanism of rumenitis remains unclear. Therefore, the aim of this study was to investigate the molecular mechanism of rumenitis in dairy cows with SARA.

**Results:**

The results showed that SARA cows displayed high concentrations of ruminal volatile fatty acids, lactic acid and lipopolysaccharide (LPS). Furthermore, the blood concentrations of LPS and acute phase proteins haptoglobin, serum amyloid-A, and LPS binding protein were significantly higher in SARA cows than in control cows. Importantly, the phosphorylation levels of nuclear factor-kappaB (NF-κB) p65, inhibitor of NF-κB (IκB), c-Jun N-terminal kinase (JNK), and extracellular signal-regulated kinase 1/2 (ERK1/2) were significantly higher in the rumen epithelium of SARA cows than those of control cows. The ruminal mRNA and protein levels of NF-κB- and mitogen-activated protein kinase (MAPK)s -regulated inflammatory cytokines, tumor necrosis factor α (TNF-α), interleukin 6 (IL-6) and interleukin 1β (IL-1β), were markedly higher in SARA cows than in control cows. Similarly, serum concentrations of TNF-α and IL-6 were also significantly higher in SARA cows.

**Conclusions:**

These results indicate that SARA results in high concentration of ruminal LPS, which over activates the NF-κB and MAPKs inflammatory pathways and then significantly increases the expression and synthesis of pro-inflammation cytokines in the rumen epithelium, thereby partly inducing rumenitis.

## Background

Subacute ruminal acidosis (SARA) is a metabolic disease in high-producing dairy cattle. This disease is caused by feeding high concentrate diets and is defined as a depression of ruminal pH below 5.6 at least 3 h/day [1]. Study in the United States has been indicated that up to 19% of early lactation dairy cows as well as 26% of mid-lactation cows have SARA [2]. This disease affects feed intake, milk production, rumen microflora, rumen digestion, and can cause diarrhea, rumen mucosal damage, laminitis, rumenitis, and liver abscesses in dairy cows [3–6].

Rumen acidosis can induce rumenitis, but the inflammatory pathogenesis is not well characterized. It has been suggested that low rumen pH could result in death and lysis of gram-negative bacteria and hence increase free lipopolysaccharide (LPS) in the rumen [7]. This increase accompanies the augmented LPS binding protein (LBP) concentration [8]. LBP is a specific acute phase protein, and is considered as a marker to evaluate inflammatory response in cattle [8]. Furthermore, haptoglobin (Hp) and serum amyloid-A (SAA) is also considered as inflammatory markers in cattle. Their concentrations in blood have been shown to increase as a result of SARA [1, 9].

Ruminal LPS is an inducer for inflammation and is associated with the development of rumenitis. Among the many signaling pathways that respond to inflammatory response, the nuclear factor-kappaB (NF-κB) pathway and mitogen-activated protein kinase (MAPK) family members are crucial signal transduction mediators that respond to LPS [10, 11]. Normally, the transcription factor NF-κB is sequestered in the cytoplasm bound to its inhibitor IκBα. Once stimulated by some stimuli, NF-κB unit p65 separates from inhibitor of NF-κB (IκB) and translocates into the nucleus where NF-κB can regulate the transcription of several inflammatory cytokines genes, such as tumor necrosis factor alpha (TNF-α), interleukin 6 (IL-6), and interleukin 1 beta (IL-1β). Three subfamilies of MAPKs have been identified: c-Jun N-terminal kinase (JNK), extracellular signal-regulated kinase 1/2 (ERK1/2), and p38 mitogen-activated protein kinase (p38MAPK), and are deemed as stress responsive and thus involved in inflammation [12]. Nevertheless, the expression changes of these inflammatory pathways were not investigated in the rumen epithelium of cows with SARA. The ruminal LPS content was significantly increased in cows with SARA [13]. Accordingly, we hypothesized that over activation of NF-κB and MAPKs inflammatory pathways induced by LPS resulted in the inflammation response of rumen epithelium in SARA cows.

## Methods

### Animals

The Ethics Committee on the Use and Care of Animals at Jilin University approved the study protocol (Changchun, China). Ten multiparous mid-lactating Holstein cows fitted with ruminal fistula were randomly assigned into SARA group (5 cows) and control group (5 cows). These Holstein cows were selected from a 3000-cow dairy farm located in Suihua City, Heilongjiang Province, China. Cows averaged 539 ± 12.6 kg of body weight and with an average milk yield of 27 ± 1.6 kg/d (mean ± SD) at the beginning of the experiment. The SARA model was prepared for induced by Khafipour et al. (2009b) [14] and Jin et al. (2016) [15]. The component of ingredients, nutrient composition, and forage to concentrate ratio (F:C) of the total mixed ratio (TMR) and wheat-barley pellets (WBP) were also according to the study of Khafipour et al. (2009b) [14]. The experiment was continued 8 wks. In control group, cows were fed with TMR with an F:C of 50:50 during the 8 wks. In SARA group, cows were fed with TMR with an F:C of 50:50 during wk. 1 to 3. A SARA challenge was conducted by increased the ratio of concentrate to an F:C of 40:60 from wk. 4 to 8. Cows were fed two times daily and had unlimited access to fresh water throughout the experiment. After this study, these cows have been kept for our other studies about SARA. During the experimental work, the cows were housed in a climate-controlled barn in individual tie stalls to reduce environmental effects.

### Rumen pH measurement

Rumen pH was monitored continuously during the experiment in 5 ruminally cannulated cows (SARA group) using indwelling pH probes as described by Gozho et al. (2006) [12]. The pH probes were placed in the ventral sac of the rumen. Rumen pH was measured at 1-min intervals and data stored in a data logger for subsequent analysis as described by Gozho et al. (2006) [12]. The purpose of ruminal pH measurement is to evaluate the success of high concentrate-induced SARA models. The pH data were summarized as average pH and time spent below pH 5.6 for each 24 h period. SARA was defined that rumen pH was below 5.6 for at least 3 h/day. The average pH in SARA group was 5.95 and the duration with pH below 5.6 was 6.15 h/day. However, the average pH in control group was 6.51 and the duration was 0.6 h/day. The rumen fluid, rumen epithelium and blood samples were collected at 8w.

### Rumen fluid sampling and analysis

Rumen fluid samples were collected from all cows at 15 min before feeding and 6 h after feeding. We opened the rumen fistula cap. A 50-mL centrifuge tube was invaded into the rumen liquid phase and the rumen fluid was collected. In ruminally cannulated cows, rumen fluid was sampled from the ventral sac of the rumen. Ruminal contents were filtered through four layers of cheesecloth. Briefly, rumen fluid samples were centrifuged at 10,000×*g* for 45 min at 4 °C. The supernatant was aspirated gently to prevent its mixing with the pellet and passed through a disposable 0.22-μm LPS-free filter. The filtrate was collected in a sterile tube for subsequent LPS measurement. LPS concentration was determined using a chromogenic Limulus amoebocyte lysate (LAL) endpoint assay (CE64406; Xiamen BioEndo Technology, Co. Ltd., Xiamen, China) according to the manufacturer’s instructions.

The rumen fluid sample was immediately centrifuged at 2500×*g* for 15 min. The supernatant was used to determine the concentrations of volatile fatty acids (VFA; acetate, propionate, butyrate, valerate, isobutyrate, and isovalerate) and lactate by gas chromatography (Model 3400 Star, Varian, Walnut Creek, CA) as described by Khafipour et al. (2009a) [4].

### The determination of blood parameters

Blood samples were collected by tail venipuncture from each cow at 15 min before feeding and 6 h after feeding. The blood samples were collected in blank and heparinized 10-mL evacuated tubes for serum and plasma collection, respectively. The concentration of LPS in plasma was determined by a chromogenic kinetic LAL assay (CE32545; Xiamen BioEndo Technology, Co. Ltd.) with a minimum detection limit of 0.005 EU/mL according to the manufacturer’s instructions. The plasma levels of β-hydroxybutyrate (BHB) and glucose were determined using an automatic biochemistry analyzer with commercially available kits (Randox Laboratories, Beijing, China).

The serum concentrations of Hp and plasma concentrations of SAA and LBP were determined using enzyme-linked immunosorbent assay (ELISA) kits (Hp: ml002480; SAA: ml002466; LBP: ml024581; Shanghai Enzyme-linked Biotechnology Co., Ltd., Shanghai, China), respectively. Samples were initially diluted 1:1 for Hp and SAA and 1:5 for LBP and assayed according to the manufacturer’s instructions. Samples were analyzed and absorbance values were read at 450 nm for Hp, SAA, and LBP using a spectrophotometer (Thermo Scientific Instrument Inc., Shanghai, China). Furthermore, the serum concentrations of TNF-α, IL-6, and IL-1β were also determined using ELISA kits (TNF-α: ml024586; IL-6: ml023756; IL-1β: ml023753; Shanghai Enzyme-linked Biotechnology Co., Ltd.) according to the manufacturer’s instructions, respectively.

### Quantitative real-time polymerase chain reaction (qRT-PCR) assay

A part of rumen content was moved out through rumen fistula to facilitate the retraction of the ventral sac. Approximately 1 g rumen papillae were excised at the ventral sac by an experienced surgical veterinarian.. The ruminal epithelium samples were washed 10 times by ice-cold saline, and were stored in the − 80 °C freezer and for the RNA and protein extraction. The ruminal incision was closed immediately. The total RNA was extracted from 0.2 g rumen epithelium using RNAiso Plus (TaKaRa Biotechnology Co., Ltd., Dalian, China) according to the manufacturer’s instructions. RNA concentration and quality was detected using an RNA/DNA calculator (Cambridge, UK) and electrophoresis (1% agarose gels), respectively. Then, total RNA in each sample was reverse transcribed to cDNA using a reverse transcription kit (TaKaRa Biotechnology Co. Ltd.), according to the supplier’s protocol. mRNA expression levels were evaluated using qRT-PCR with the SYBR Green QuantiTect RT-PCR Kit (TaKaRa Biotechnology Co., Ltd.) and a 7500 Real-Time PCR System (Applied Biosystems Inc.). The relative expression of each gene was normalized to β-actin. The primers for each gene were shown in Table 1.

**Table 1:** The primers sequences used for cDNA generation

Gene	Sequence number	Primer sequences (5′-3′)	Length (bp)
TNFα	NW_003104557.1	For CTGCCGGACTACCTGGACTAT Rev. CCTCACTTCCCTACATCCCTAA	234
IL6	NW_00310889.1	For AACGAGTGGGTAAAGAACGC Rev. CTGACCAGAGGAGGGAATGC	144
IL1β	NW_003104294.1	For CTGAACCCATCAACGAAA Rev. ATGACCGACACCACCTGC	190
β-actin	BC 142413.1	For GCCCTGAGGCTCTCTTCCA Rev. GCGGATGTCGACGTCACA	101

### Western blotting assay

The total protein was extracted from 0.5 g ruminal epithelium according to the manufacturer’s instructions (Sangon Biotech Co., Ltd., Shanghai, China). Thus, protein concentration was determined using a protein assay reagent (Beyotime Biotechnology Inc., China). Target proteins were separated by sodium dodecyl sulfate-polyacrylamide gel electrophoresis (SDS-PAGE) and then electrophoretically transferred to a polyvinylidene difluoride (PVDF) membrane. Next, the membranes were blocked in 3% bovine serum albumin (BSA)-Tris-buffered saline-Tween buffer for 4 h. The blocked membranes were hybridized overnight at 4 °C with primary antibodies (Abcam, Cambridge, UK; Cell Signaling Technology, Danvers, MA, USA), respectively. After being washed 4 times, the blocked membranes were incubated in a horseradish peroxidase (HRP)-conjugated secondary antibody at room temperature for 45 min. Immunoreactive bands were visualized by enhanced chemiluminescence solution (ECL, Beyotime Biotechnology Inc., Shanghai, China). Finally, the bands were imaged using a protein simple imager (ProteinSimple, Santa Clara, CA, USA).

### Statistical analysis

Data are expressed as the means ± standard deviation (SD) and analyzed using SPSS (Statistical Package for the Social Sciences) 16.0 software (SPSS Incorporated, Chicago, IL, USA). Differences among groups were compared with Student’s test. A *P* value < 0.05 was considered statistically significant and *P* value < 0.01 was marked significant compared to control group.

## Results

### The rumen levels of VFA, lactic acid, and LPS

As shown in the Table 2, the rumen content of total VFA (*P* <  0.01), acetate (*P* <  0.01), propionate (*P* < 0.05), butyrate (*P* < 0.05), isobutyrate (*P* < 0.05), and valerate (*P* < 0.05) were significantly higher in cows with SARA than in control cows. Importantly, the ruminal lactic acid and LPS concentrations in SARA cows was 3.6 times and 4.2 times as great as the control cows (*P* < 0.01), respectively. These results indicate that cows with SARA display ruminal VFA, lactic acid, and LPS accumulation.

**Table 2:** The concentration of ruminal and blood parameters in the control and SARA cows

Item	Control (*n* = 5)	SARA (*n* = 5)	*P*-value
Total Ruminal VFA, *mM*	93.6 ± 5.6	130.9 ± 6.2**	0.001
Acetate, *mM*	66.2 ± 4.1	88.3 ± 4.5**	0.008
Propionate, *mM*	15.5 ± 2.9	23.5 ± 2.6*	0.021
Butyrate, *mM*	10.9 ± 1.7	17.1 ± 2.6*	0.024
Isobutyrate*, mM*	2.0 ± 0.3	2.8 ± 0.2*	0.036
Valerate*, mM*	1.7 ± 0.2	2.4 ± 0.3*	0.033
LPS, EU/mL	30,768 ± 1035	130,589 ± 1675**	0.004
Lactic acid*, mM*	0.95 ± 0.2	3.38 ± 0.4**	0.000
Plasma BHBA, *mM*	0.62 ± 0.03	0.66 ± 0.02	0.129
Plasma glucose, *mM*	3.87 ± 0.11	3.94 ± 0.12	0.632
Plasma LPS, EU/mL	< 0.005	0.21 ± 0.06**	0.000

* *P* < 0.05, ** *P* < 0.01 versus the control group

### Blood parameters

**Fig. 1:**
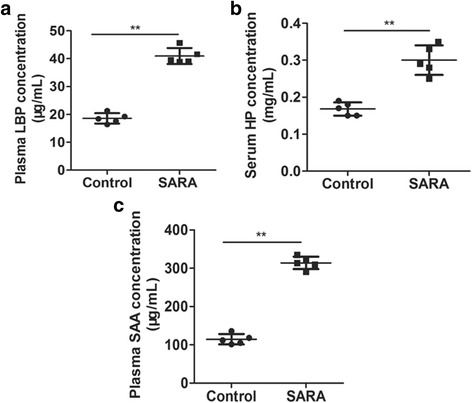
The blood levels of LBP, Hp, and SAA in the control and SARA cows. Five cows were in each group. **a** The plasma level of LBP; **b** The serum level of Hp; **c** The plasma level of SAA. The data presented are the mean ± SD. * *P* < 0.05 and ** *P* < 0.01 versus the control group

As shown in Table 2, the blood concentrations of glucose and BHB were slightly higher in the SARA cows than in the control cows, but there were no significant statistical difference (*P* > 0.05). Plasma LPS concentration in control cows was below the minimum detection level of 0.005 EU/mL for the method. Following the SARA challenge, the average LPS in plasma was 0.21 EU/mL. The blood concentrations of the acute phase proteins LBP, Hp, and SAA in SARA group were 40.9 μg/mL, 0.300 mg/mL, and 313.9 μg/mL, and were significantly higher than those of 18.6 μg/mL, 0.168 mg/mL, and 114.5 μg/mL in control group, respectively (Fig. 1, *P* < 0.01), which suggested that cows with SARA displayed severe inflammation response.

### MAPKs and NF-κB inflammation pathways were over induced in the ruminal epithelium of cows with SARA

**Fig. 2:**
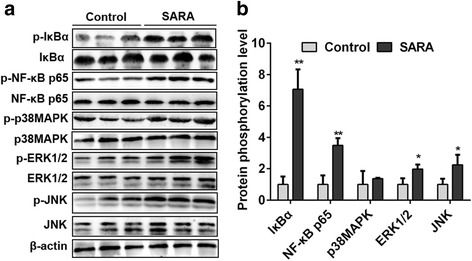
The phosphorylation levels of NF-κB and MAPKs in the ruminal epithelium of control and SARA cows. Five cows were in each group. **a** The western blotting results of p-IκBα, IκBα, p-NF-κB p65, NF-κB p65, p-p38MAPK, p38MAPK, p-ERK, ERK, p-JNK, JNK, and β-actin; B: The phosphorylation levels of IκBα, NF-κB p65, p38MAPK, ERK1/2 and JNK. The data presented are the mean ± SD. * *P* < 0.05, ** *P* < 0.01 versus the control group

To investigate the mechanism of rumenitis, the inflammatory pathways MAPKs and NF-κB were detected using Western Blotting. As shown in Fig. 2, the phosphorylation levels of IκBα (p-IκBα/IκBα) and NF-κB p65 (p-NF-κB p65/NF-κB p65) were significantly higher in cows with SARA than in control cows (*P* < 0.01). Furthermore, the phosphorylation levels of JNK (p-JNK/JNK) and ERK1/2 (p-ERK1/2/ERK1/2) were significantly increased in cows with SARA (*P* < 0.05). In addition, p38MAPK (p-p38MAPK/p38MAPK) phosphorylation level was slightly higher in SARA cows than in control cows (*P* > 0.05). Taken together, these data indicate that MAPKs and NF-κB inflammation pathways are over induced in the ruminal epithelium of cows with SARA.

### The mRNA and protein expression levels of inflammatory cytokines

**Fig. 3:**
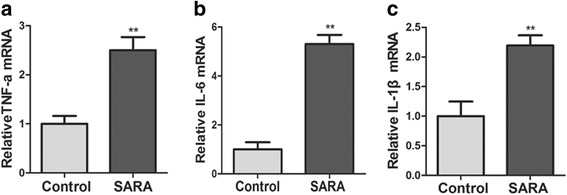
The mRNA expression levels of inflammatory cytokines in the ruminal epithelium of control and SARA cows. Five cows were in each group. **a** The mRNA expression levels of *TNF-α*; **b** The mRNA expression levels of *IL-6*; (C) The mRNA expression levels of *IL-1β*. The data presented are the mean ± SD. * *P* < 0.05, ** *P* < 0.01 versus the control group

**Fig. 4:**
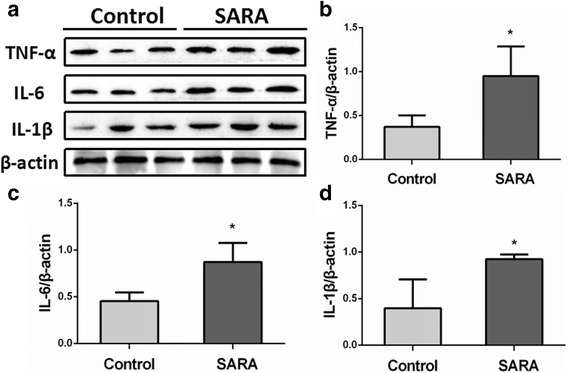
The protein expression levels of inflammatory cytokines in the ruminal epithelium of control and SARA cows. Five cows were in each group. **a** The western blotting results of TNF-α, IL-6, and IL-1β; **b** The protein expression levels of TNF-α; **c** The protein expression levels of IL-6; **d** The protein expression levels of IL-1β. The data presented are the mean ± SD. * *P* < 0.05, ** *P* < 0.01 versus the control group

MAPKs and NF-κB pathways over activation could promote the transcription expression of genes coded inflammatory cytokines [16]. In this study, the mRNA and protein expression levels of TNF-α, IL-6, and IL-1β in the ruminal epithelium of cows with SARA were significantly higher than those of control cows (Fig. 3, *P* < 0.01; Fig. 4, *P* < 0.05).

### The blood concentrations of inflammatory cytokines

**Fig. 5:**
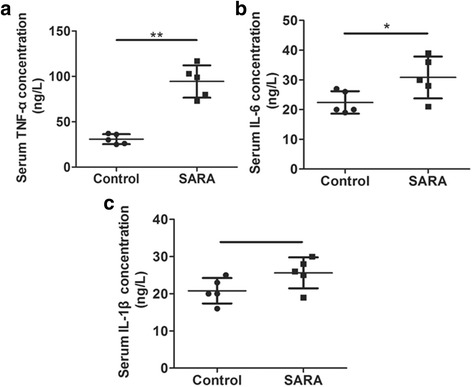
The blood concentrations of inflammatory cytokines in the control and SARA cows. Five cows were in each group. **a**The blood concentration of TNF-α; **b** The blood concentration of IL-6; **c** The blood concentration of IL-1β. The data presented are the mean ± SD. * *P* < 0.05, ** *P* < 0.01 versus the control group

As shown in Fig. 5, the blood concentrations of TNF-α and IL-6 was significantly higher in SARA cows than in control cows (*P* < 0.01, *P* < 0.05). Furthermore, the IL-1β concentrations were higher in SARA cows than in control cows, but there were no significant statistical difference (*P* > 0.05). These results further demonstrated that high expression of TNF-α, IL-6, and IL-1β further mediated the inflammation response of ruminal epithelium in SARA cows.

## Discussion

SARA is a common health problem in dairy cattle. Suddenly switching cattle from a high-forage to a high-starch diet result in a decrease of ruminal pH that is characteristic of SARA because VFA accumulates in the rumen [17]. Furthermore, the increase of high-starch diet of cattle during gradual grain adaptation results in microbiological changes in the rumen. If the rate of concentrate inclusion in the diet is higher than the rate at which lactate-utilizing bacteria can increase, then lactic acid accumulates in the rumen and depresses rumen pH more drastically than similar amounts of VFA [18]. In this study, we found the content of total VFA, acetate, propionate, butyrate, and lactic acid in rumen fluid were significantly higher in SARA cows than in control cows. Our data further indicated that SARA appeared to be caused by an elevation in total VFA and lactic acid, which further resulted in a significant decrease in ruminal pH.

During low rumen pH, gram-negative bacteria lyse more rapidly, increasing the concentration of LPS in the rumen [7]. In our experiment, the ruminal content of LPS was 130,589 EU/mL in grain-induced SARA cows and significantly higher than that of 30,768 EU/mL in control cows signi. This change was greater than the reported ranges for grain-induced SARA, which was from 3715 to 12,589 EU/mL, 24,547 to 128,820 EU/mL, from 28,184 to 107,150 EU/mL, reported by Gozho et al. (2005) [1], Gozho et al. (2007) [3] and Khafipour et al. (2009a) [4], respectively. Furthermore, Rodríguez-Lecompte et al. (2014) reported that ruminal LPS content of cows with grain-based SARA was up to 168,391 EU/mL [6], which was greater than our data. The discrepancy between our study and previous studies is probably because of differences in the body condition, nutrient composition, the method of LPS determination, which affects the ruminal LPS production. The presence of LPS could result in the production of multiple proinflammatory cytokines [15] and cause massive disruption of ruminal epithelial tight junctions [19]. Rumen wall damage associated with SARA further increased ruminal LPS translocation into the bloodstream [4, 5, 14]. Khafipour et al. (2009b) reported for the first time that a grain-based SARA challenge increased the LPS from < 0.05 (Control) to 0.52 EU/mL (SARA) [14]. In addition, Jin et al. (2016) also reported that the plasma LPS concentration of lacteal artery and vein in cows with long-term high-concentrate diet feeding was 0.86 and 0.27 EU/mL, respectively [15]. In our study, the plasma LPS concentration was 0.21 EU/mL in SARA cows. However, many studies did not detect the LPS in peripheral circulation during experimentally induced ruminal acidosis [6, 20]. This discrepancy may be due to the difference of damage degree of rumen wall in SARA cows.

When LPS released in large quantities, these mediators induced an acute phase response. Hp, SAA, and LBP are such proteins that are used as inflammatory markers in cattle [8, 21]. In the present study, the blood Hp, SAA and LBP concentrations were markedly increased in cows with SARA, which indicated that cows with SARA displayed high inflammatory levels. Many previous studies also demonstrated that blood Hp, SAA and LBP concentrations were significantly increased in cows with SARA or grain-induced SARA modal [1, 4, 14, 22, 23], which further supported our data.

Rumenitis is the direct result of SARA in dairy cows. As the degradation of ruminal environment, ruminal epithelium was continuously exposed to high levels of LPS in SARA cows. LPS is an inflammatory inducer and may induce the development of rumenitis. Zhang et al. (2016) also reported that LPS involved in the development of inflammation of cows with SARA [24]. MAPKs and NF-κB pathways are the main inflammatory pathways that mediate the LPS challenge. Over activation of NF-κB, JNK, p38MAPK and ERK1/2 were also observed in the mastitis induced by LPS [25]. In this study, the phosphorylation levels of IκBα and NF-κB were significantly increased in rumen epithelium of cows with SARA than in control cows. This indicated that NF-κB pathway was involved in the development of rumenitis. Interestingly, Fan et al. (2016) found that NF-κB was involved in the LPS-mediated proliferation and apoptosis of MAC-T epithelial cells as part of the SARA response in cows, which further supported our results in vitro [26]. In addition, we found that JNK and ERK1/2 phosphorylation levels were markedly increased in the rumen epithelium of SARA cows. Over activation of MAPKs and NF-κB inflammatory pathways could significantly increase the expression of inflammatory cytokines [25]. As expected, our results showed that the mRNA and protein levels of TNF-α, IL-6 and IL-1β in ruminal epithelium and the blood concentrations of TNF-α and IL-6 were significantly higher in SARA cows than in control cows. These results indicate that MAPKs and NF-κB inflammatory pathways are over-activated and further induce the overproduction of inflammatory cytokines in ruminal epithelium. These inflammatory cytokines further mediated the inflammation response in ruminal epithelium through a paracrine fashion, thereby partly inducing rumenitis in cows with SARA. Zhang et al. (2016) reported that high-concentrate feeding upregulated the expression of inflammation-related genes IL-1β, IL-2 and IL-22 in the ruminal epithelium of dairy cattle [24]. They also found that LPS treatment significantly increased the mRNA expression of IL-1β, IL-2, IL-6 and IL-8 in ruminal epithelial cells of Holstein cows in vitro. These studies further demonstrated that over activation of inflammatory pathways mediated the development of rumenitis in SARA cows.

## Conclusion

In summary, our results suggest that SARA-induced high levels of LPS over activate the MAPKs and NF-κB inflammatory pathways and increase the production of inflammatory cytokines in ruminal epithelium, thereby inducing rumenitis at least in part. These findings will promote new exploration into the prophylaxis and treatment of SARA in dairy cows.

## Abbreviations

ERK1/2: Extracellular signal-regulated kinase 1/2
Hp: Haptoglobin
IL-1β: Interleukin 1β
IL-6: Interleukin 6
JNK: c-Jun N-terminal kinase
LAL: Limulus amoebocyte lysate
LBP: LPS binding protein
LPS: Lipopolysaccharide
MAPK: Mitogen-activated protein kinase
NF-κB: Nuclear factor-kappaB
p38MAPK: p38 mitogen-activated protein kinase
PVDF: Polyvinylidene difluoride
qRT-PCR: Quantitative real-time polymerase chain reaction
SAA: Serum amyloid-A
SARA: Subacute ruminal acidosis
SDS-PAGE: Sodium dodecyl sulfate-polyacrylamide gel electrophoresis
TNF-α: Tumor necrosis factor α
